# Does typing of *Chlamydia trachomatis* using housekeeping multilocus sequence typing reveal different sexual networks among heterosexuals and men who have sex with men?

**DOI:** 10.1186/s12879-016-1486-2

**Published:** 2016-04-18

**Authors:** Bart Versteeg, Sylvia M. Bruisten, Arie van der Ende, Yvonne Pannekoek

**Affiliations:** Public Health Laboratory, Cluster Infectious Diseases, Public Health Service Amsterdam, Amsterdam, The Netherlands; Center for Infection and Immunity Amsterdam (CINIMA), Academic Medical Center, University of Amsterdam, Amsterdam, The Netherlands; Department of Medical Microbiology, Academic Medical Center, Amsterdam, The Netherlands

**Keywords:** *Chlamydia trachomatis*, Multilocus sequence typing, MLST, High-resolution genotyping, Sexually transmitted infections, MSM

## Abstract

**Background:**

*Chlamydia trachomatis* infections remain the most common bacterial sexually transmitted infection worldwide. To gain more insight into the epidemiology and transmission of *C. trachomatis*, several schemes of multilocus sequence typing (MLST) have been developed. We investigated the clustering of *C. trachomatis* strains derived from men who have sex with men (MSM) and heterosexuals using the MLST scheme based on 7 housekeeping genes (MLST-7) adapted for clinical specimens and a high-resolution MLST scheme based on 6 polymorphic genes, including *ompA* (hr-MLST-6).

**Methods:**

Specimens from 100 *C. trachomatis* infected men who have sex with men (MSM) and 100 heterosexual women were randomly selected from previous studies and sequenced. We adapted the MLST-7 scheme to a nested assay to be suitable for direct typing of clinical specimens. All selected specimens were typed using both the adapted MLST-7 scheme and the hr-MLST-6 scheme. Clustering of *C. trachomatis* strains derived from MSM and heterosexuals was assessed using minimum spanning tree analysis.

**Results:**

Sufficient chlamydial DNA was present in 188 of the 200 (94 %) selected samples. Using the adapted MLST-7 scheme, full MLST profiles were obtained for 187 of 188 tested specimens resulting in a high success rate of 99.5 %. Of these 187 specimens, 91 (48.7 %) were from MSM and 96 (51.3 %) from heterosexuals. We detected 21 sequence types (STs) using the adapted MLST-7 and 79 STs using the hr-MLST-6 scheme. Minimum spanning tree analyses was used to examine the clustering of MLST-7 data, which showed no reflection of separate transmission in MSM and heterosexual hosts. Moreover, typing using the hr-MLST-6 scheme identified genetically related clusters within each of clusters that were identified by using the MLST-7 scheme.

**Conclusion:**

No distinct transmission of *C. trachomatis* could be observed in MSM and heterosexuals using the adapted MLST-7 scheme in contrast to using the hr-MLST-6. In addition, we compared clustering of both MLST schemes and demonstrated that typing using the hr-MLST-6 scheme is able to identify genetically related clusters of *C. trachomatis* strains within each of the clusters that were identified by using the MLST-7 scheme.

**Electronic supplementary material:**

The online version of this article (doi:10.1186/s12879-016-1486-2) contains supplementary material, which is available to authorized users.

## Background

Despite intensive efforts to reduce the spread of chlamydial infections, *Chlamydia trachomatis* remains the primary cause of bacterial sexually transmitted diseases worldwide [1]. These *C. trachomatis* infections can result in serious sequelae including epididymitis and pelvic inflammatory disease, leading to infertility in women and possibly also in men [2–4].

Molecular epidemiological studies are essential to understand the genetic population structure and to gain insight into the transmission of *C. trachomatis* [5, 6]. Until recently, the epidemiology of *C. trachomatis* was based on serotyping of the major outer membrane protein (MOMP) or sequence analysis of its encoding gene *ompA*. However, typing based on the *ompA*-gene only is not recommended as typing method for *C. trachomatis,* as whole-genome-sequencing (WGS) data revealed that it is an unstable and unreliable target due to extensive recombination [7]. Numerous sets of WGS data have become available for a variety of bacterial isolates including *C. trachomatis* [7]. Unfortunately, due to the complex intracellular lifecycle of *C. trachomatis* it remains technically challenging to perform WGS directly on clinical specimens. Instead, several multilocus sequence typing (MLST) schemes were successfully developed and validated to gain insight into the epidemiology and transmission of *C. trachomatis* [8–11]. An advantage of using MLST schemes to type *C. trachomatis* strains, is that it is a standardized and portable method to index variation among strains, which can easily be used in different laboratories on a global basis since it yields robust data [12–14].

In previous studies a clear separation of *C. trachomatis* strain types was seen for specimens derived from men who have sex with men (MSM) and from heterosexuals as defined by a high-resolution MLST scheme based on 6 polymorphic genes, including *ompA* (herein referred to as hr-MLST-6) [11, 15–17]. The hr-MLST-6 scheme was specifically designed for short-term epidemiology and outbreak investigations and was intended to be highly discriminating. In contrast, another MLST scheme was designed based on 7 housekeeping genes (herein referred to as MLST-7) [9]. The slow rate of molecular evolution within these housekeeping genes makes the MLST-7 scheme useful to answer evolutionary questions and to investigate the epidemiology of *C. trachomatis* over a longer time-frame. A limitation of the published MLST-7 scheme is that it consists of single PCRs, which has a decreased sensitivity making it less suitable for typing of *C. trachomatis* strains from direct clinical specimens and is therefore not applicable for large population studies. Typing of *C. trachomatis* using the MLST-7 scheme is therefore often dependent on an additional culture step possibly leading to biased study outcomes. Moreover, it is unknown whether the clear separation in transmission of *C. trachomatis* strain types for specimens derived from MSM and from heterosexuals can also be seen using the MLST-7 scheme.

The objective of this study was therefore to: (1) adapt the MLST-7 scheme to a nested assay to be suitable for direct typing of clinical specimens without the need for an additional cell culture step, (2) determine whether separate *C. trachomatis* transmission in MSM and from heterosexual hosts could also be seen using the MLST-7 scheme and (3) compare clustering of the *C. trachomatis* strain types according to both MLST schemes.

## Methods

### Clinical specimen selection

For this retrospective analysis, *C. trachomatis-*positive specimens and data were selected from a previous study [17]. All specimens and data were matched on sexgroup and a random selection was made using SPSS 21 (SPSS Inc., Chicago, IL, USA). In total, 100 specimens from MSM and 100 specimens from heterosexual women were selected. Rectal swab specimens from MSM were collected between July 2008 and August 2009. *C. trachomatis*-positive urogenital swab specimens from heterosexual women were collected between December 2011 and December 2012.

### Ethics

The STI outpatient clinic of the Public Health Service of Amsterdam, the Netherlands uses an opting-out approach to recruit clients for scientific research. All clients of the STI clinic are notified that remainders of samples may be used for scientific research, after anonymisation and de-identification of client clinical data and samples. If clients objected, data and samples are discarded. Therefore, no additional informed consent was obtained for this study. This procedure was approved by the Medical Ethical Committee of the Academic Medical Center of the University of Amsterdam, the Netherlands (reference number W14-200 # 14.17.0247).

### DNA Extraction and amplification

For DNA extraction 200 μl swab-containing transport medium of specimens that tested positive in a screening assay (Aptima combo TMA test, Hologic/Gen-Probe, San Diego, California, USA) was added to 500 μl lysis buffer (bioMérieux, Boxtel, the Netherlands), 1 μg glycogen (10 mg/ml; Roche Diagnostics, Almere, the Netherlands). DNA was subsequently precipitated by adding 700 μl of ice-cold isopropanol. The DNA precipitate was washed twice with 70 % ethanol and dissolved in 50 μl 10 mM Tris-buffer (pH 8.0). These DNA isolates were stored at −20 °C until further use.

Extracted DNA was tested to indicate the load of chlamydial DNA using an in-house *pmpH* real time PCR [11, 16, 17]. For DNA specimens that tested *pmpH* negative, DNA was re-extracted from the original Aptima *C. trachomatis* positive specimens and retested. All specimens that repeatedly tested negative for the *pmpH* target were excluded.

### Primer selection for adaptation of MLST-7

Primers were designed and a nested PCR assay was developed to enable direct testing of clinical samples. MLST-7 regions were analyzed *in silico* using the Chlamydiales MLST database (http://pubmlst.org/chlamydiales/) Comparison of all available *C. trachomatis* sequences from the MLST database demonstrated that shortening of the original amplicons for 5 out of the 7 genes (*gatA, oppA_3, hflX, gidA* and *fumC*) to perform nested PCR would not result in loss of resolution as no known polymorphic sites for *C. trachomatis* were found in these ‘deleted’ regions. Therefore, new ‘inner’ PCR primers were designed for these 5 genes (Table 1). For two other genes (*hemN* and *enoA*) the original amplicon was extended to prevent loss of resolution and known polymorphic sites. Therefore, new ‘outer’ PCR primers were designed, thus the same inner PCR product was obtained as published previously (Table 1) [9].

**Table 1:** Primers used for the MLST-7 scheme for Chlamydia trachomatis^a^

Region	Locus tag^b^	Format	Direction	Sequence (5′ to 3′)	5′ position^c^	Fragment length (bp)^d^
*gatA*	CT0003	**Outer**	**Forward**	**GCTTTAGAATTARSARAWGCT**	2123	325
		**Outer**	**Reverse**	**GATCCTCCGGTATCYGATCC**	2615	
		Inner + Seq	Forward	ATGACGAACAGATTGGAGC	2186	
		Inner + Seq	Reverse	GGATTATTGGTAGGATGAA	2532	
*oppA_3*	CT0198	**Outer**	**Forward**	**ATGCGCAAGATATCAGTGGG**	222438	480
		**Outer**	**Reverse**	**AAAGCTCCRSTWGMTATMGGWAG**	223002	
		Inner + Seq	Forward	TCCTAGCATTAGCAACTTCT	222469	
		Inner + Seq	Reverse	TCTTTCCGTATCTGATGCTGCG	222970	
*hflX*	CT0379	**Outer**	**Forward**	**GCTTCTARAGTACTTTTAAATG**	432737	359
		**Outer**	**Reverse**	**TATTTRGAAATYTTTKCSAGYCG**	432758	
		Inner + Seq	Forward	AAGTATGCGGAAGTTTGCG	432768	
		Inner + Seq	Reverse	AATCAGGAGGTAGTGGTGGAGG	433106	
*gidA*	CT0498	**Outer**	**Forward**	**GGAGTCWCTACWAAAGAAGG**	577352	389
		**Outer**	**Reverse**	**TCGTAYTGYACATCRAAAGG**	577892	
		Inner + Seq	Forward	ACTTCTCTGGGGGACGATT	577449	
		Inner + Seq	Reverse	GACCGTTCACATAAACTTCTTG	577856	
*enoA*	CT0587	Outer	Forward	GCAAATACTTTACAGAGACCTT	662222	388
		Outer	Reverse	CGTCACAAATAGGTCGTCTC	662775	
		**Inner + Seq**	**Forward**	**CCTATGATGAATCTKATCAATGG**	662288	
		**Inner + Seq**	**Reverse**	**TCTTCTTCGGCTAGCCCATCT**	662698	
*hemN*	CT0746	Outer	Forward	GAATCTTGCCTTTCACAGTTGC	867674	448
		Outer	Reverse	ACTTCCACATCCCATTCTGC	868584	
		**Inner + Seq**	**Forward**	**AGATCTTCTTCWGGRGGWAGAGA**	867799	
		**Inner + Seq**	**Reverse**	**TTCYTTCAKAACSTAGGTTTT**	868269	
*fumC*	CT0855	**Outer**	**Forward**	**ATTAAAAAATGTGCTGCT**	1004691	451
		**Outer**	**Reverse**	**CCTTCAGGAACATTYAACCC**	1005242	
		Inner + Seq	Forward	ATTAAAAAATGTGCTGCT	1004691	
		Inner + Seq	Reverse	CCGCTCTAAACAATTATGCAACTG	1005159	

^a^The primers given in boldface were the original MLST primers taken from Pannekoek et al. [9]. All other primers were newly designed. Nested PCRs were performed for each region. The primers used for the outer PCRs are indicated with Outer, those used for the inner PCRs are indicated with Inner, and those used for sequencing are indicated with Seq

^b^Locus tags are given relative to the sequence of reference strain D/UW-3/CX (GenBank accession no. AE001273)

^c^Positions are given in base pairs relative to the sequence of reference strain D/UW-3/CX (GenBank accession no. AE001273)

^d^Fragment length is the number of base pairs sequenced with the primers excluded

### Nested PCR of MLST regions

For MLST-7, DNA extracts were amplified by nested PCR for all regions using the oligonucleotide primers shown in Table 1. Full hr-MLST-6 data was already available for all specimens from previous studies [17]. Also for this typing method a nested PCR was used to amplify the regions *ompA*, CT046 (*hctB*), CT058, CT144, CT172, and CT682 (*pbpB*). For both MLST schemes the inner PCR was performed with M13-tagged primers, identical to the standard inner primers. This allowed sequencing using universal M13 primers for high throughput purposes [11, 16, 17].

### Sequencing analysis

The obtained sequences were analysed, assembled and trimmed using BioNumerics 7.5 (Applied Maths, Sint-Martens-Latem, Belgium). For MLST-7, cleaned primer-to-primer sequences were checked against the Chlamydiales MLST database (http://pubmlst.org/chlamydiales/). For hr-MLST-6, analysis was performed as described previously [17]. In brief, cleaned primer-to-primer sequences were checked against the *Chlamydia trachomatis* hr-MLST database (http://mlstdb.bmc.uu.se/).

### Minimum spanning tree analysis

BioNumerics software (version 7.5, Applied Maths, Sint-Martens-Latem, Belgium) was used to construct a minimum spanning tree of fully typed specimens using STs of each of the MLST schemes. As algorithm we used the predefined template ‘Minimum spanning tree for categorical data’ plugin to generate an minimum spanning tree under the categorical coefficient of similarity and the priority rule of the highest number of single-locus variants (SLV), which calculates a standard minimum spanning tree. A large cluster was defined as a group of genetically related STs differing by not more than 1 locus from another ST within that group (SLV) and it had to include at least 5 % of the total number of specimens. Clusters containing less then 5 % of the total number of specimens were defined as small clusters.

## Results

### *C. trachomatis* typing using the MLST-7 scheme

Although in 188 of 200 *C. trachomatis*-positive specimens (94.0 %), sufficient chlamydial DNA was present, as assessed by qPCR, no amplicons were obtained using the MLST scheme, primers and cycle conditions as were previously described [9], indicating relative low sensitivity of the method. We therefore adapted the MLST-7 scheme to a nested assay and, using this adapted MLST-7 scheme, for 187 (93.5 %) specimens full MLST-7 profiles were obtained. Of these 187 typed specimens, 91 (48.7 %) specimens were from MSM and 96 (51.3 %) specimens were from heterosexuals (Additional file 1: Table S1). For the 187 fully typed specimens, we found 7 novel allele sequences: 3 for *oppA_*3, 1 for *gidA*, 1 for *enoA*, and 2 for *hemN*.

Among the 187 typed specimens, 21 unique MLST-7 STs could be assigned of which 18 (85.7 %) were novel to the Chlamydiales MLST database (http://pubmlst.org/chlamydiales/). Novel STs were found in 125 of 187 specimens (66.8 %). Of all identified STs, 8 (38.1 %) had multiple representatives (2 to 52 isolates) while 13 (61.9 %) were found in only a single isolate each (Table 2). Moreover, 4 STs (ST97, ST132, ST133, ST134; 19.0 %) were found in specimens from both MSM and heterosexuals and 6 STs (ST44, ST97 ST132, ST133, ST137, ST142; 28.6 %) were associated with multiple (2 to 5) *ompA* genovars (Table 2).

**Table 2:** Results of *C. trachomatis* using the MLST-7 scheme based on seven housekeeping genes in comparison with *ompA*-typing and sexgroup

Sequence type	Number of strains	*gatA*	*oppA_3*	*hflX*	*gidA*	*enoA*	*hemN*	*fumC*	Cluster	*ompA* genovar (Number of strains)	Sexgroup^a^ (number of strains)
8	1	2	1	1	2	4	2	3	B	F (1)	Hetero (1)
44	18	1	3	3	3	2	2	19	C	L2 (1), L2b (17)	MSM (18)
97	42	33	1	2	2	4	2	3	B	D (24), E(1), F(17)	Hetero (17), MSM (25)
106	1	2	3	2	5	3	2	3	A	J (1)	Hetero (1)
131	1	33	27	2	4	3	2	3	A	G (1)	MSM (1)
132	52	33	3	2	4	3	2	3	A	G (26), H (1), I (10), J (13), K(2)	Hetero (13), MSM (39)
133	26	33	1	1	2	4	2	3	B	D (3), E (21), F (2)	Hetero (20), MSM (6)
134	2	33	1	1	2	3	2	3	B	F (2)	Hetero (1), MSM (1)
135	19	33	4	1	2	4	2	3	B	E (19)	Hetero (19)
136	11	33	3	2	5	3	2	3	A	B (1), D (2), G (4), J (4)	Hetero (11)
137	1	33	3	2	40	3	2	3	A	G (1)	Hetero (1)
138	1	33	29	1	2	4	2	3	B	E (1)	Hetero (1)
139	1	33	1	1	2	32	2	3	B	E (1)	Hetero (1)
140	1	33	3	2	4	3	1	3	A	G (1)	Hetero (1)
141	4	33	3	2	5	3	1	3	A	D (3), I (1)	Hetero (4)
142	1	33	4	1	32	4	2	3	B	E (1)	Hetero (1)
143	1	33	3	2	5	3	25	3	A	K (1)	Hetero (1)
144	1	33	3	2	5	3	24	3	A	G (1)	Hetero (1)
145	1	33	3	2	4	24	2	3	A	H (1)	Hetero (1)
146	1	2	4	1	2	4	2	3	B	F (1)	Hetero (1)
147	1	33	28	2	4	3	2	3	A	J (1)	MSM (1)

^a^ *Hetero* heterosexual, *MSM* men who have sex with men

### *C. trachomatis* typing using hr-MLST-6 scheme

Full hr-MLST-6 profiles were available for the 187 *C. trachomatis*-positive specimens that were also successfully typed using the adapted MLST-7 scheme (Additional file 2: Table S2). The hr-MLST-6 data from the 187 *C. trachomatis*-positive specimens have been described before in more detail [11, 15–17]. In brief, among the 187 typed specimens, 79 unique hr-MLST-6 STs could be assigned of which 22 (27.8 %) had multiple representatives (2 to 18 isolates) while 57 (72.2 %) were found in only a single isolate each (Table 3). Of all identified STs, 3 (ST12d, ST56a, ST90; 3.8 %) were found in specimens from both MSM and heterosexuals (Table 3).

**Table 3:** Results of *C. trachomatis* typing using the hr-MLST-6 scheme based on six polymorphic genes in comparison with *ompA*-typing and sexgroup

Sequence type	Number of strains	*ompA*	CT046	CT058	CT144	CT172	CT682	Cluster	*ompA* genovar (number of strains)	Sexgroup^a^ (number of strains)
3	10	6	1	2	6	2	2	VII	E (10)	Hetero (10)
11	3	1	5	19	7	2	10		D (3)	MSM (3)
16	1	6	7	19	14	2	1	VI	E (1)	Hetero (1)
27	1	9	10	6	10	1	6		G (1)	Hetero (1)
30	1	12	10	7	1	3	8		K (1)	MSM (1)
32	1	12	10	7	1	4	8		K (1)	Hetero (1)
33	6	8	10	8	5	3	6	II	G (6)	MSM (6)
35	3	2	10	8	1	4	17		D (3)	Hetero (3)
52	17	8	20	8	5	3	6	II	G (17)	MSM (17)
59	1	6	7	19	7	2	1	VI	E (1)	Hetero (1)
69	1	6	5	19	6	2	2		E (1)	Hetero (1)
74	1	30	8	8	1	7	18		B (1)	Hetero (1)
90	2	24	5	19	1	1	4	IV	F (2)	Hetero (1), MSM (1)
101	1	36	38	5	12	7	18	III	I (1)	Hetero (1)
109	18	1	5	20	5	2	34	V	D (18)	MSM (18)
110	1	24	1	2	7	2	4	IV	F (1)	Hetero (1)
137	1	8	10	8	22	4	6		G (1)	Hetero (1)
143	1	28	44	13	17	13	28	VIII	L2b (1)	MSM (1)
153	2	6	35	19	7	2	1	VI	E (2)	Hetero (2)
165	1	35	12	5	1	9	8		H (1)	Hetero (1)
171	2	6	49	19	7	2	1	VI	E (2)	Hetero (2)
172	1	6	1	2	7	2	2	VII	E (1)	Hetero (1)
194	3	1	5	6	5	2	34	V	D (3)	MSM (3)
232	2	20	5	19	1	4	18		J (2)	Hetero (2)
240	1	24	5	19	12	1	4	IV	F (1)	Hetero (1)
265	1	10	10	4	1	3	7		G (1)	Hetero (1)
270	1	8	10	5	12	3	8		G (1)	Hetero (1)
272	1	36	10	5	12	24	18	III	I (1)	Hetero (1)
288	1	8	10	8	22	7	57		G (1)	Hetero (1)
301	1	8	20	8	5	1	6	II	G (1)	MSM (1)
303	1	8	20	8	5	3	34	II	G (1)	MSM (1)
304	1	8	20	8	5	4	6	II	G (1)	MSM (1)
305	1	6	25	2	6	2	2	VII	E (1)	Hetero (1)
307	2	53	29	8	5	3	58	I	J (2)	MSM (2)
318	1	1	45	20	5	2	34	V	D (1)	MSM (1)
324	1	1	55	20	5	2	34	V	D (1)	MSM (1)
338	1	6	67	2	7	2	60		E (1)	Hetero (1)
341	1	6	70	57	7	2	1		E (1)	Hetero (1)
345	2	53	74	8	5	3	6	I	J (2)	MSM (2)
353	1	8	81	8	5	3	6	II	G (1)	MSM (1)
395	1	2	83	4	1	3	17		D (1)	Hetero (1)
435	1	9	8	6	10	1	6		G (1)	Hetero (1)
448	1	6	1	2	11	2	2	VII	E (1)	Hetero (1)
449	1	6	1	19	1	1	1		E (1)	Hetero (1)
450	1	24	1	19	7	2	4	IV	F (1)	Hetero (1)
453	1	6	1	19	7	21	1	VI	E (1)	Hetero (1)
459	1	6	5	19	6	21	2		E (1)	Hetero (1)
462	1	6	5	19	7	2	56		E (1)	Hetero (1)
465	1	6	5	19	7	14	2		E (1)	Hetero (1)
466	1	24	5	19	7	21	4	IV	F (1)	Hetero (1)
482	1	9	10	6	10	1	8		G (1)	Hetero (1)
484	1	37	10	7	1	3	5		I (1)	Hetero (1)
498	1	6	24	19	14	2	1	VI	E (1)	Hetero (1)
502	1	6	45	19	6	1	2		E (1)	Hetero (1)
504	1	6	47	19	7	2	1	VI	E (1)	Hetero (1)
509	1	6	71	2	1	2	2	VII	E (1)	Hetero (1)
510	1	6	71	2	6	2	2	VII	E (1)	Hetero (1)
513	2	6	71	19	7	2	1	VI	E (2)	Hetero (2)
516	1	6	5	19	5	2	2		E (1)	Hetero (1)
517	2	36	38	5	12	3	18	III	I (2)	Hetero (2)
100b	3	36	10	5	12	7	18	III	I (3)	Hetero (3)
100c	1	37	10	5	12	7	18	III	I (1)	Hetero (1)
108c	9	53	29	8	5	3	6	I	J (9)	MSM (9)
12d	13	24	5	19	7	1	4	IV	F (13)	Hetero (12), MSM (1)
135a	1	20	10	5	12	4	18	III	J (1)	Hetero (1)
135b	2	36	10	5	12	4	18	III	I (2)	Hetero (2)
13b	1	24	5	19	15	1	4	IV	F (1)	Hetero (1)
148a	2	24	5	19	7	2	4	IV	F (2)	Hetero (1), MSM (1)
205b	1	20	10	6	22	4	8		J (1)	Hetero (1)
20a	1	2	10	4	1	4	17		D (1)	Hetero (1)
220a	1	12	10	4	1	3	8		K (1)	Hetero (1)
281a	1	20	10	8	1	4	18		J (1)	Hetero (1)
281b	1	63	10	8	1	4	18		J (1)	Hetero (1)
56a	9	6	1	19	7	2	1	VI	E (9)	Hetero (7), MSM (2)
58a	1	22	27	13	17	13	28	VIII	L2 (1)	MSM (1)
58b	16	28	27	13	17	13	28	VIII	L2b (16)	MSM (16)
77b	1	31	5	19	7	2	37		D (1)	Hetero (1)
91a	1	24	5	19	5	2	4	IV	F (1)	MSM (1)
97b	1	35	12	5	11	9	8		H (1)	Hetero (1)

^a^ *Hetero* heterosexual, *MSM* men who have sex with men

### Minimum spanning tree analysis of the MLST-7 scheme and the hr-MLST-6 scheme

**Fig. 1:**
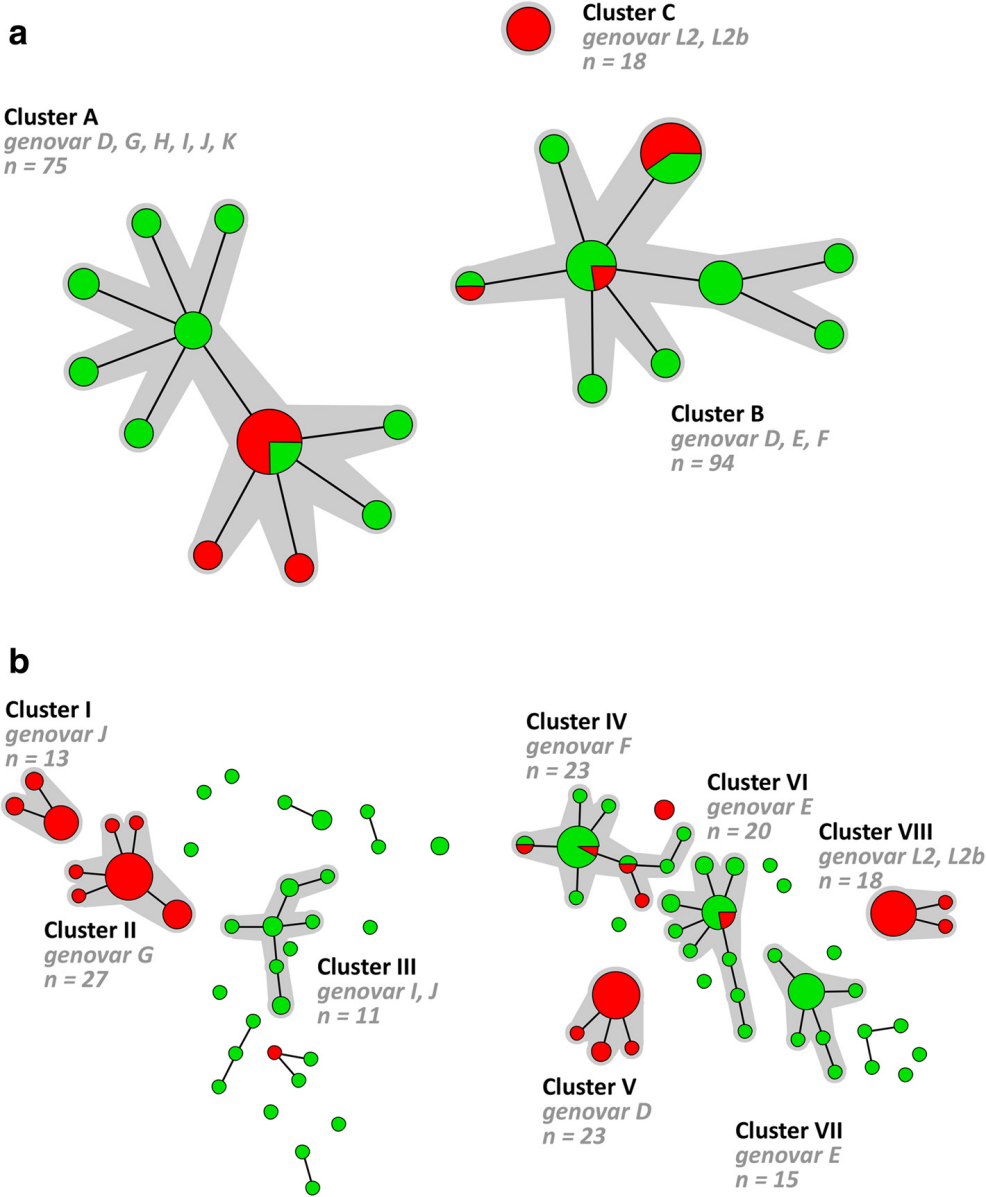
Minimum spanning tree showing the clustering of 187 *Chlamydia trachomatis*-positive specimens from MSM and heterosexuals. Each circle represents one ST. Size of the circles is proportional to the number of identical ST profiles. Bold lines connect types that differ for one single locus. Halos indicate the distinct clusters. **a** Minimum spanning tree showing the clustering of the *C. trachomatis*-positive specimens according to the MLST-7 scheme; **b** Minimum spanning tree showing the clustering of the *C. trachomatis*-positive specimens according to the hr-MLST-6 scheme. The colour coding is: red, men who have sex with men (*n* = 91); green, heterosexuals (*n* = 96)

A minimum spanning tree was generated based on the MLST-7 STs and 3 large clusters (A-C) could be identified (Fig. 1a). The number of specimens in these clusters ranged from 18 to 94 and included all typed specimens so no single isolates (singletons) or small clusters were identified. The minimum spanning tree showed mixed clusters of specimens from MSM and heterosexual individuals and a low genetic diversity among the *C. trachomatis* population. Cluster A contained 41 specimens from MSM (54.7 %) that were associated with 3 STs (Table 2). Two of those STs (ST131, ST148) were found in one MSM specimen each, whereas the remaining ST (ST132) was found in specimens from 52 individuals of whom 39 (75.0 %) where MSM. Cluster B contained 32 specimens from MSM (34.0 %) that were associated with 3 STs (ST97, ST133, ST134). Of those, ST97 was found in 42 specimens from individuals of whom 25 (59.5 %) were MSM, ST133 was found in specimens from 26 individuals of whom 6 were MSM and ST134 was found in specimens from 2 individuals of whom 1 was MSM. Cluster C solely consisted of specimens from MSM which were all associated with the same ST (ST44).

A minimum spanning tree was also generated basted on the hr-MLST-6 STs and 8 large clusters (I-VIII) were identified (Fig. 1b). These clusters ranged from 13 to 27 specimens comprising 80.2 % of all specimens. The remaining 37 specimens were distributed over 20 singletons and 6 small clusters, ranging from 2 to 4 specimens. The minimum spanning tree showed a clear distinction between specimens from MSM and heterosexual individuals and high genetic diversity among the *C. trachomatis* population especially for strains from heterosexuals, as was also previously described by us [11, 15–17]. Of the 8 large clusters, 4 (III, IV, VI and VII) consisted predominantly of specimens from heterosexuals (82.6 to 100 %) whereas the other 4 large clusters (I, II, V and VIII) solely consisted of specimens from MSM (Table 3). Of the 4 clusters (III, IV, VI and VII) consisting predominantly of specimens from heterosexuals, only 2 clusters (IV and VI) contained MSM specimens. Cluster IV contained 4 specimens from MSM (17.4 %) that were associated with 4 STs (ST12d, ST90, ST91a). Of those 4 STs, ST12d was found in specimens from 13 individuals of whom 1 was MSM, ST90 was found in specimens from 2 individuals of whom 1 was MSM and ST91a was found in only one MSM specimen. Cluster VI contained 2 specimens from MSM which were associated with 1 ST (ST56a). This ST was found in specimens from 9 individuals of whom 2 were MSM.

**Fig. 2:**
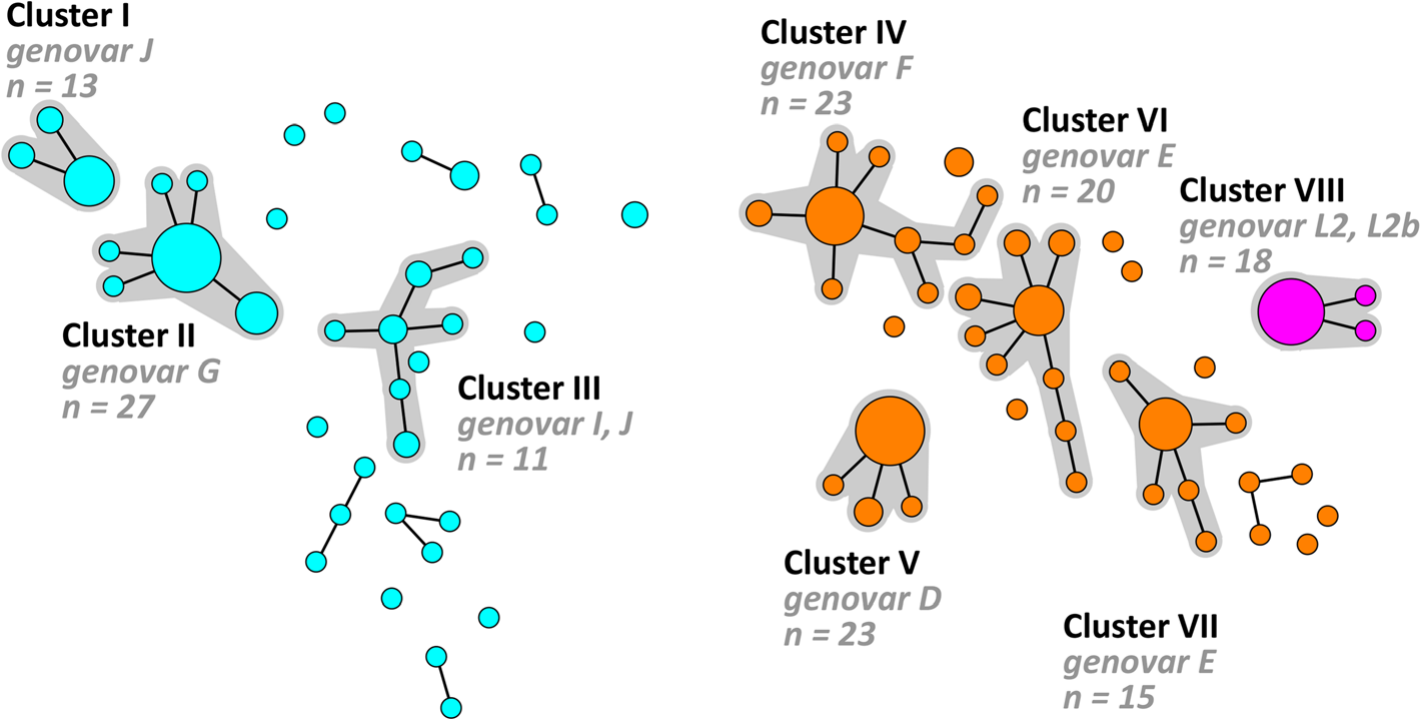
Minimum spanning tree showing the diversification by hr-MLST-6 of the clusters that were generated using the MLST-7 scheme. Each circle represents one ST. Size of the circles is proportional to the number of identical ST profiles. Bold lines connect types that differ for one single locus. Halos indicate the distinct clusters. Colours indicate the MLST-7 clusters from Fig. 1a: blue, cluster A (*n* = 75); orange, cluster B (*n* = 94); fuchsia, cluster C (*n* = 18)

Comparison of the two minimum spanning trees in Fig. 1 shows that the hr-MLST-6 scheme further diversified each of the clusters generated by using the adapted MLST-7 scheme. For example cluster A (11 STs; Fig. 1a) is subdivided into clusters I, II, and III, using the hr-MLST-6 scheme, and also the majority of the singletons and small clusters (Fig. 2) comprising 37 STs. Cluster B (9 STs; Fig. 1a) is further subdivided into clusters IV, V, VI, VII and some remaining singletons and small clusters (Fig. 2) comprising 39 STs. Cluster C (1 ST; Fig. 1a) is identical to cluster VIII (Figs. 1b and 2) but included 3 STs.

## Discussion

In this study, we successfully adapted the MLST-7 scheme to a nested assay and applied it to direct clinical specimens. Moreover, we demonstrated that using this adapted MLST-7 scheme no reflection of separate transmission between MSM and heterosexual hosts was seen. In addition, we compared clustering of both typing methods and demonstrated that typing using the hr-MLST-6 scheme is able to identify small genetically related clusters of *C. trachomatis* strains within each of the clusters that were identified by using the modified MLST-7 scheme (Fig. 2). These clusters seem to represent biovars.

We successfully modified the MLST-7 scheme that was previously published by us [9]. The sensitivity of the MLST-7 method was increased by modification into a nested PCR format. This nested PCR format allowed detection of many novel sequence types because we were now able to test direct clinical specimens without the need for an additional cell culture step. In total, 187 of 188 samples could be fully typed using the modified MLST-7 scheme resulting in a high success rate of 99.5 %. Failure to generate an MLST-7 profile for one out of 188 samples after repeated testing is most likely the result of an insufficient amount of chlamydial DNA in this sample as it produced the highest cycling threshold value in the *pmpH* real time PCR.

The discriminatory capacity of the original MLST-7 scheme was maintained as no known polymorphic sites for *C. trachomatis* were lost due to shortening of some of the amplicons. Moreover for two genes (*hemN* and *enoA*) the amplicon was extended because new ‘outer’ PCR primers were designed which ensured that the same inner PCR product was obtained as published previously [9]. Designing ‘inner’ PCR primers for these two genes would have resulted in very short amplicons and loss of resolution as known polymorphic sites would have been lost. The maximum target length in our assay format was 480 bp, which allowed easy sequence analysis from single PCR fragments. It is unlikely that polymorphisms were missed by our adapted MLST-7 scheme. Even if there were so far unknown missed polymorphisms, we think that these would not dramatically increase the genetic diversity using the adapted MLST-7 scheme.

The MLST-7 scheme was developed according to the original MLST principle [13, 18, 19], which aims to index the diversity of nucleotide sequences of fragments of housekeeping genes. Housekeeping genes are presumed to be under neutral or nearly neutral selection pressure making them stable over time, which makes this method useful to answer evolutionary questions and to investigate the epidemiology of *C. trachomatis* over a longer period in time [18]. In comparison, the hr-MLST-6 scheme uses non-housekeeping genes that are under immune pressure or have variable repeat regions and are thus more polymorphic [11, 16]. This enabled us to demonstrate detailed genetic differences between *C. trachomatis* strains that were involved in transmission chains in human hosts in a short time-frame of only a few years [11, 15–17]. However, using the adapted MLST-7 scheme no reflection of separate transmission networks between MSM and heterosexual hosts was seen. Minimum spanning tree analysis of the MLST-7 scheme showed mixed clusters of specimens from MSM and heterosexual individuals with the exception of one cluster (Fig. 1a; cluster C) which solely included specimens from MSM. All these cluster C *C. trachomatis* specimens belonged to the lymphogranuloma venereum (LGV) biovar, which is known to show major genetic differences compared to the urogenital biovar types [20–23]. These results suggest that separate transmission chains in human hosts can only be observed over a small time-frame using highly discriminating genetic typing methods. Transmission chains between MSM and heterosexuals were not totally separated in our previous studies with larger trees, including randomly chosen samples [15–17]. This overlap was expected since there will also be individuals who identify themselves as MSM, but have had contact with heterosexual women (Fig. 1b).

Comparison of the clustering of both MLST schemes showed that both MLST schemes were able to resolve the *C. trachomatis* specimens in a number of STs and clusters. Minimum spanning tree analysis using data from the adapted MLST-7 scheme revealed 3 main groups of circulating *C. trachomatis* strains (Fig. 1), as was also previously described by us using the original MLST-7 scheme [9]. One group is composed solely of the LGV genovars. Another is composed of the clinically prevalent urogenital genovars, whereas the third contains the less frequently occurring urogenital genovars. Similar groupings are reported in previous studies using WGS analysis or MLST analysis based on housekeeping genes [7, 9, 10]. In comparison to MLST-7, minimum spanning tree analysis of the hr-MLST-6 scheme was able to further diversify each of these 3 main groups and clusters of circulating *C. trachomatis* strains in smaller genetically related clusters and thus allowed for more detailed analysis within these groups (Fig. 2). Using maximum likelihood analysis, we previously demonstrated that the hr-MLST-6 targets provided a tree similar to trees based on WGS, but with lower bootstrap support values [12]. Since we already observed clear differences in the diversification of strains between the MLST-7 and hr-MLST-6 scheme (Fig. 1), a similar tree based on the MLST-7 targets is expected to differ from a tree based on WGS by showing less genetic diversity.

## Conclusions

We successfully adapted and applied the MLST-7 scheme to direct clinical specimens from MSM and heterosexuals. However, using this adapted MLST-7 scheme no distinct transmission of *C. trachomatis* could be observed in MSM and heterosexuals in comparison to the hr-MLST-6 scheme. In addition, we also compared clustering of both MLST schemes and demonstrated that typing using the hr-MLST-6 scheme is able to identify genetically related clusters of *C. trachomatis* strains within each of the clusters that were identified by using the MLST-7 scheme. Hr-MLST-6 may therefore also be a useful tool to for more detailed analysis of *C. trachomatis* strains within identified MLST-7 clusters.

### Availability of data and materials

Data on all 187 samples typed with both the hr-MLST-6 en MLST-7 scheme are included in Additional file 1: Table S1 and Additional file 2: Table S2. Corresponding nucleotide sequences can be derived from the publicly available databases: http://mlstdb.bmc.uu.se/ (hr-MLST-6) and http://pubmlst.org/chlamydiales/ (MLST-7).

## Additional files

Additional file 1: Table S1. MLST-7 data of the 187 successfully typed samples. (DOCX 57 kb) Additional file 2: Table S2. Hr-MLST-6 data of the 187 successfully typed samples. (DOCX 53 kb)
