# Supplementary material for: Does typing of Chlamydia trachomatis using housekeeping multilocus sequence typing reveal different sexual networks among heterosexuals and men who have sex with men?
Source: BMC Infect Dis. 2016 Apr 18;16:162. doi: 10.1186/s12879-016-1486-2 (PMC4836166; doi:10.1186/s12879-016-1486-2)
Supplement: Additional file 1: Table S1. — MLST-7 data of the 187 successfully typed samples. (DOCX 57 kb) [file 12879_2016_1486_MOESM1_ESM.docx]

**Supplementary Table 1. MLST-7 data of the 187 successfully typed samples.**

Coding is according to the MLST-7 database (http://pubmlst.org/chlamydiales/). The samples are sorted by cluster and sequence type.

| **Cluster name** | **ID** | **Sequence type** | **Sexgroup^a^** | ***ompA* genovar** | ***gatA*** | ***oppA_3*** | ***hflX*** | ***gidA*** | ***enoA*** | ***hemN*** | ***fumC*** |
| --- | --- | --- | --- | --- | --- | --- | --- | --- | --- | --- | --- |
| Cluster A | P187 | 106 | Hetero | J | 2 | 3 | 2 | 5 | 3 | 2 | 3 |
|  | P050 | 131 | MSM | G | 33 | 27 | 2 | 4 | 3 | 2 | 3 |
|  | P006 | 132 | MSM | G | 33 | 3 | 2 | 4 | 3 | 2 | 3 |
|  | P007 | 132 | MSM | J | 33 | 3 | 2 | 4 | 3 | 2 | 3 |
|  | P008 | 132 | MSM | J | 33 | 3 | 2 | 4 | 3 | 2 | 3 |
|  | P009 | 132 | MSM | G | 33 | 3 | 2 | 4 | 3 | 2 | 3 |
|  | P014 | 132 | MSM | G | 33 | 3 | 2 | 4 | 3 | 2 | 3 |
|  | P016 | 132 | MSM | J | 33 | 3 | 2 | 4 | 3 | 2 | 3 |
|  | P020 | 132 | MSM | G | 33 | 3 | 2 | 4 | 3 | 2 | 3 |
|  | P023 | 132 | MSM | G | 33 | 3 | 2 | 4 | 3 | 2 | 3 |
|  | P024 | 132 | MSM | G | 33 | 3 | 2 | 4 | 3 | 2 | 3 |
|  | P025 | 132 | MSM | G | 33 | 3 | 2 | 4 | 3 | 2 | 3 |
|  | P026 | 132 | MSM | G | 33 | 3 | 2 | 4 | 3 | 2 | 3 |
|  | P028 | 132 | MSM | J | 33 | 3 | 2 | 4 | 3 | 2 | 3 |
|  | P030 | 132 | MSM | G | 33 | 3 | 2 | 4 | 3 | 2 | 3 |
|  | P031 | 132 | MSM | G | 33 | 3 | 2 | 4 | 3 | 2 | 3 |
|  | P033 | 132 | MSM | G | 33 | 3 | 2 | 4 | 3 | 2 | 3 |
|  | P037 | 132 | MSM | G | 33 | 3 | 2 | 4 | 3 | 2 | 3 |
|  | P038 | 132 | MSM | J | 33 | 3 | 2 | 4 | 3 | 2 | 3 |
|  | P040 | 132 | MSM | G | 33 | 3 | 2 | 4 | 3 | 2 | 3 |
|  | P048 | 132 | MSM | G | 33 | 3 | 2 | 4 | 3 | 2 | 3 |
|  | P049 | 132 | MSM | J | 33 | 3 | 2 | 4 | 3 | 2 | 3 |
|  | P051 | 132 | MSM | J | 33 | 3 | 2 | 4 | 3 | 2 | 3 |
|  | P054 | 132 | MSM | J | 33 | 3 | 2 | 4 | 3 | 2 | 3 |
|  | P055 | 132 | MSM | G | 33 | 3 | 2 | 4 | 3 | 2 | 3 |
|  | P059 | 132 | MSM | G | 33 | 3 | 2 | 4 | 3 | 2 | 3 |
|  | P061 | 132 | MSM | G | 33 | 3 | 2 | 4 | 3 | 2 | 3 |
|  | P065 | 132 | MSM | J | 33 | 3 | 2 | 4 | 3 | 2 | 3 |
|  | P066 | 132 | MSM | G | 33 | 3 | 2 | 4 | 3 | 2 | 3 |
|  | P069 | 132 | MSM | G | 33 | 3 | 2 | 4 | 3 | 2 | 3 |
|  | P070 | 132 | MSM | G | 33 | 3 | 2 | 4 | 3 | 2 | 3 |
|  | P071 | 132 | MSM | J | 33 | 3 | 2 | 4 | 3 | 2 | 3 |
|  | P072 | 132 | MSM | J | 33 | 3 | 2 | 4 | 3 | 2 | 3 |
|  | P074 | 132 | MSM | G | 33 | 3 | 2 | 4 | 3 | 2 | 3 |
|  | P075 | 132 | MSM | J | 33 | 3 | 2 | 4 | 3 | 2 | 3 |
|  | P079 | 132 | MSM | K | 33 | 3 | 2 | 4 | 3 | 2 | 3 |
|  | P080 | 132 | MSM | G | 33 | 3 | 2 | 4 | 3 | 2 | 3 |
|  | P082 | 132 | MSM | G | 33 | 3 | 2 | 4 | 3 | 2 | 3 |
|  | P085 | 132 | MSM | G | 33 | 3 | 2 | 4 | 3 | 2 | 3 |
|  | P086 | 132 | MSM | G | 33 | 3 | 2 | 4 | 3 | 2 | 3 |
|  | P087 | 132 | MSM | G | 33 | 3 | 2 | 4 | 3 | 2 | 3 |
|  | P094 | 132 | Hetero | K | 33 | 3 | 2 | 4 | 3 | 2 | 3 |
|  | P096 | 132 | Hetero | I | 33 | 3 | 2 | 4 | 3 | 2 | 3 |
|  | P099 | 132 | Hetero | I | 33 | 3 | 2 | 4 | 3 | 2 | 3 |
|  | P106 | 132 | Hetero | I | 33 | 3 | 2 | 4 | 3 | 2 | 3 |
|  | P116 | 132 | Hetero | I | 33 | 3 | 2 | 4 | 3 | 2 | 3 |
|  | P119 | 132 | Hetero | I | 33 | 3 | 2 | 4 | 3 | 2 | 3 |
|  | P123 | 132 | Hetero | I | 33 | 3 | 2 | 4 | 3 | 2 | 3 |
|  | P131 | 132 | Hetero | I | 33 | 3 | 2 | 4 | 3 | 2 | 3 |
|  | P139 | 132 | Hetero | I | 33 | 3 | 2 | 4 | 3 | 2 | 3 |
|  | P142 | 132 | Hetero | J | 33 | 3 | 2 | 4 | 3 | 2 | 3 |
|  | P165 | 132 | Hetero | I | 33 | 3 | 2 | 4 | 3 | 2 | 3 |
|  | P181 | 132 | Hetero | I | 33 | 3 | 2 | 4 | 3 | 2 | 3 |
|  | P183 | 132 | Hetero | H | 33 | 3 | 2 | 4 | 3 | 2 | 3 |
|  | P101 | 136 | Hetero | G | 33 | 3 | 2 | 5 | 3 | 2 | 3 |
|  | P113 | 136 | Hetero | B | 33 | 3 | 2 | 5 | 3 | 2 | 3 |
|  | P115 | 136 | Hetero | D | 33 | 3 | 2 | 5 | 3 | 2 | 3 |
|  | P137 | 136 | Hetero | G | 33 | 3 | 2 | 5 | 3 | 2 | 3 |
|  | P140 | 136 | Hetero | J | 33 | 3 | 2 | 5 | 3 | 2 | 3 |
|  | P143 | 136 | Hetero | J | 33 | 3 | 2 | 5 | 3 | 2 | 3 |
|  | P150 | 136 | Hetero | D | 33 | 3 | 2 | 5 | 3 | 2 | 3 |
|  | P153 | 136 | Hetero | J | 33 | 3 | 2 | 5 | 3 | 2 | 3 |
|  | P163 | 136 | Hetero | G | 33 | 3 | 2 | 5 | 3 | 2 | 3 |
|  | P178 | 136 | Hetero | J | 33 | 3 | 2 | 5 | 3 | 2 | 3 |
|  | P184 | 136 | Hetero | G | 33 | 3 | 2 | 5 | 3 | 2 | 3 |
|  | P179 | 137 | Hetero | G | 33 | 3 | 2 | 40 | 3 | 2 | 3 |
|  | P114 | 140 | Hetero | G | 33 | 3 | 2 | 4 | 3 | 1 | 3 |
|  | P107 | 141 | Hetero | D | 33 | 3 | 2 | 5 | 3 | 1 | 3 |
|  | P129 | 141 | Hetero | I | 33 | 3 | 2 | 5 | 3 | 1 | 3 |
|  | P149 | 141 | Hetero | D | 33 | 3 | 2 | 5 | 3 | 1 | 3 |
|  | P176 | 141 | Hetero | D | 33 | 3 | 2 | 5 | 3 | 1 | 3 |
|  | P144 | 143 | Hetero | K | 33 | 3 | 2 | 5 | 3 | 25 | 3 |
|  | P146 | 144 | Hetero | G | 33 | 3 | 2 | 5 | 3 | 24 | 3 |
|  | P147 | 145 | Hetero | H | 33 | 3 | 2 | 4 | 24 | 2 | 3 |
|  | P017 | 147 | MSM | J | 33 | 28 | 2 | 4 | 3 | 2 | 3 |
| Cluster B | P100 | 8 | Hetero | F | 2 | 1 | 1 | 2 | 4 | 2 | 3 |
|  | P001 | 97 | MSM | D | 33 | 1 | 2 | 2 | 4 | 2 | 3 |
|  | P002 | 97 | MSM | D | 33 | 1 | 2 | 2 | 4 | 2 | 3 |
|  | P003 | 97 | MSM | D | 33 | 1 | 2 | 2 | 4 | 2 | 3 |
|  | P010 | 97 | MSM | D | 33 | 1 | 2 | 2 | 4 | 2 | 3 |
|  | P012 | 97 | MSM | D | 33 | 1 | 2 | 2 | 4 | 2 | 3 |
|  | P021 | 97 | MSM | D | 33 | 1 | 2 | 2 | 4 | 2 | 3 |
|  | P022 | 97 | MSM | D | 33 | 1 | 2 | 2 | 4 | 2 | 3 |
|  | P029 | 97 | MSM | D | 33 | 1 | 2 | 2 | 4 | 2 | 3 |
|  | P034 | 97 | MSM | D | 33 | 1 | 2 | 2 | 4 | 2 | 3 |
|  | P036 | 97 | MSM | D | 33 | 1 | 2 | 2 | 4 | 2 | 3 |
|  | P039 | 97 | MSM | D | 33 | 1 | 2 | 2 | 4 | 2 | 3 |
|  | P041 | 97 | MSM | F | 33 | 1 | 2 | 2 | 4 | 2 | 3 |
|  | P044 | 97 | MSM | D | 33 | 1 | 2 | 2 | 4 | 2 | 3 |
|  | P047 | 97 | MSM | D | 33 | 1 | 2 | 2 | 4 | 2 | 3 |
|  | P053 | 97 | MSM | D | 33 | 1 | 2 | 2 | 4 | 2 | 3 |
|  | P057 | 97 | MSM | F | 33 | 1 | 2 | 2 | 4 | 2 | 3 |
|  | P058 | 97 | MSM | D | 33 | 1 | 2 | 2 | 4 | 2 | 3 |
|  | P060 | 97 | MSM | D | 33 | 1 | 2 | 2 | 4 | 2 | 3 |
|  | P063 | 97 | MSM | D | 33 | 1 | 2 | 2 | 4 | 2 | 3 |
|  | P064 | 97 | MSM | D | 33 | 1 | 2 | 2 | 4 | 2 | 3 |
|  | P073 | 97 | MSM | D | 33 | 1 | 2 | 2 | 4 | 2 | 3 |
|  | P077 | 97 | MSM | D | 33 | 1 | 2 | 2 | 4 | 2 | 3 |
|  | P084 | 97 | MSM | D | 33 | 1 | 2 | 2 | 4 | 2 | 3 |
|  | P088 | 97 | MSM | D | 33 | 1 | 2 | 2 | 4 | 2 | 3 |
|  | P090 | 97 | MSM | D | 33 | 1 | 2 | 2 | 4 | 2 | 3 |
|  | P097 | 97 | Hetero | F | 33 | 1 | 2 | 2 | 4 | 2 | 3 |
|  | P098 | 97 | Hetero | F | 33 | 1 | 2 | 2 | 4 | 2 | 3 |
|  | P103 | 97 | Hetero | F | 33 | 1 | 2 | 2 | 4 | 2 | 3 |
|  | P108 | 97 | Hetero | F | 33 | 1 | 2 | 2 | 4 | 2 | 3 |
|  | P120 | 97 | Hetero | F | 33 | 1 | 2 | 2 | 4 | 2 | 3 |
|  | P124 | 97 | Hetero | F | 33 | 1 | 2 | 2 | 4 | 2 | 3 |
|  | P132 | 97 | Hetero | F | 33 | 1 | 2 | 2 | 4 | 2 | 3 |
|  | P133 | 97 | Hetero | E | 33 | 1 | 2 | 2 | 4 | 2 | 3 |
|  | P135 | 97 | Hetero | F | 33 | 1 | 2 | 2 | 4 | 2 | 3 |
|  | P138 | 97 | Hetero | F | 33 | 1 | 2 | 2 | 4 | 2 | 3 |
|  | P151 | 97 | Hetero | F | 33 | 1 | 2 | 2 | 4 | 2 | 3 |
|  | P154 | 97 | Hetero | F | 33 | 1 | 2 | 2 | 4 | 2 | 3 |
|  | P156 | 97 | Hetero | F | 33 | 1 | 2 | 2 | 4 | 2 | 3 |
|  | P160 | 97 | Hetero | F | 33 | 1 | 2 | 2 | 4 | 2 | 3 |
|  | P171 | 97 | Hetero | F | 33 | 1 | 2 | 2 | 4 | 2 | 3 |
|  | P182 | 97 | Hetero | D | 33 | 1 | 2 | 2 | 4 | 2 | 3 |
|  | P185 | 97 | Hetero | F | 33 | 1 | 2 | 2 | 4 | 2 | 3 |
|  | P004 | 133 | MSM | D | 33 | 1 | 1 | 2 | 4 | 2 | 3 |
|  | P005 | 133 | MSM | D | 33 | 1 | 1 | 2 | 4 | 2 | 3 |
|  | P035 | 133 | MSM | F | 33 | 1 | 1 | 2 | 4 | 2 | 3 |
|  | P043 | 133 | MSM | D | 33 | 1 | 1 | 2 | 4 | 2 | 3 |
|  | P045 | 133 | MSM | E | 33 | 1 | 1 | 2 | 4 | 2 | 3 |
|  | P081 | 133 | MSM | E | 33 | 1 | 1 | 2 | 4 | 2 | 3 |
|  | P092 | 133 | Hetero | E | 33 | 1 | 1 | 2 | 4 | 2 | 3 |
|  | P093 | 133 | Hetero | E | 33 | 1 | 1 | 2 | 4 | 2 | 3 |
|  | P095 | 133 | Hetero | E | 33 | 1 | 1 | 2 | 4 | 2 | 3 |
|  | P104 | 133 | Hetero | E | 33 | 1 | 1 | 2 | 4 | 2 | 3 |
|  | P111 | 133 | Hetero | E | 33 | 1 | 1 | 2 | 4 | 2 | 3 |
|  | P112 | 133 | hetero | E | 33 | 1 | 1 | 2 | 4 | 2 | 3 |
|  | P118 | 133 | Hetero | E | 33 | 1 | 1 | 2 | 4 | 2 | 3 |
|  | P121 | 133 | Hetero | E | 33 | 1 | 1 | 2 | 4 | 2 | 3 |
|  | P125 | 133 | Hetero | E | 33 | 1 | 1 | 2 | 4 | 2 | 3 |
|  | P126 | 133 | Hetero | E | 33 | 1 | 1 | 2 | 4 | 2 | 3 |
|  | P127 | 133 | Hetero | E | 33 | 1 | 1 | 2 | 4 | 2 | 3 |
|  | P145 | 133 | Hetero | E | 33 | 1 | 1 | 2 | 4 | 2 | 3 |
|  | P148 | 133 | Hetero | E | 33 | 1 | 1 | 2 | 4 | 2 | 3 |
|  | P155 | 133 | Hetero | E | 33 | 1 | 1 | 2 | 4 | 2 | 3 |
|  | P157 | 133 | Hetero | E | 33 | 1 | 1 | 2 | 4 | 2 | 3 |
|  | P162 | 133 | Hetero | E | 33 | 1 | 1 | 2 | 4 | 2 | 3 |
|  | P166 | 133 | Hetero | E | 33 | 1 | 1 | 2 | 4 | 2 | 3 |
|  | P167 | 133 | Hetero | E | 33 | 1 | 1 | 2 | 4 | 2 | 3 |
|  | P170 | 133 | Hetero | E | 33 | 1 | 1 | 2 | 4 | 2 | 3 |
|  | P172 | 133 | Hetero | F | 33 | 1 | 1 | 2 | 4 | 2 | 3 |
|  | P032 | 134 | MSM | F | 33 | 1 | 1 | 2 | 3 | 2 | 3 |
|  | P175 | 134 | Hetero | F | 33 | 1 | 1 | 2 | 3 | 2 | 3 |
|  | P102 | 135 | Hetero | E | 33 | 4 | 1 | 2 | 4 | 2 | 3 |
|  | P105 | 135 | Hetero | E | 33 | 4 | 1 | 2 | 4 | 2 | 3 |
|  | P109 | 135 | Hetero | E | 33 | 4 | 1 | 2 | 4 | 2 | 3 |
|  | P117 | 135 | Hetero | E | 33 | 4 | 1 | 2 | 4 | 2 | 3 |
|  | P122 | 135 | Hetero | E | 33 | 4 | 1 | 2 | 4 | 2 | 3 |
|  | P128 | 135 | Hetero | E | 33 | 4 | 1 | 2 | 4 | 2 | 3 |
|  | P130 | 135 | Hetero | E | 33 | 4 | 1 | 2 | 4 | 2 | 3 |
|  | P136 | 135 | Hetero | E | 33 | 4 | 1 | 2 | 4 | 2 | 3 |
|  | P141 | 135 | Hetero | E | 33 | 4 | 1 | 2 | 4 | 2 | 3 |
|  | P152 | 135 | Hetero | E | 33 | 4 | 1 | 2 | 4 | 2 | 3 |
|  | P158 | 135 | Hetero | E | 33 | 4 | 1 | 2 | 4 | 2 | 3 |
|  | P159 | 135 | Hetero | E | 33 | 4 | 1 | 2 | 4 | 2 | 3 |
|  | P161 | 135 | Hetero | E | 33 | 4 | 1 | 2 | 4 | 2 | 3 |
|  | P164 | 135 | Hetero | E | 33 | 4 | 1 | 2 | 4 | 2 | 3 |
|  | P168 | 135 | Hetero | E | 33 | 4 | 1 | 2 | 4 | 2 | 3 |
|  | P173 | 135 | Hetero | E | 33 | 4 | 1 | 2 | 4 | 2 | 3 |
|  | P174 | 135 | Hetero | E | 33 | 4 | 1 | 2 | 4 | 2 | 3 |
|  | P177 | 135 | Hetero | E | 33 | 4 | 1 | 2 | 4 | 2 | 3 |
|  | P180 | 135 | Hetero | E | 33 | 4 | 1 | 2 | 4 | 2 | 3 |
|  | P186 | 138 | Hetero | E | 33 | 29 | 1 | 2 | 4 | 2 | 3 |
|  | P110 | 139 | Hetero | E | 33 | 1 | 1 | 2 | 32 | 2 | 3 |
|  | P134 | 142 | Hetero | E | 33 | 4 | 1 | 32 | 4 | 2 | 3 |
|  | P169 | 146 | Hetero | F | 2 | 4 | 1 | 2 | 4 | 2 | 3 |
| Cluster C | P011 | 44 | MSM | L2b | 1 | 3 | 3 | 3 | 2 | 2 | 19 |
|  | P013 | 44 | MSM | L2b | 1 | 3 | 3 | 3 | 2 | 2 | 19 |
|  | P015 | 44 | MSM | L2b | 1 | 3 | 3 | 3 | 2 | 2 | 19 |
|  | P018 | 44 | MSM | L2b | 1 | 3 | 3 | 3 | 2 | 2 | 19 |
|  | P019 | 44 | MSM | L2b | 1 | 3 | 3 | 3 | 2 | 2 | 19 |
|  | P027 | 44 | MSM | L2b | 1 | 3 | 3 | 3 | 2 | 2 | 19 |
|  | P042 | 44 | MSM | L2b | 1 | 3 | 3 | 3 | 2 | 2 | 19 |
|  | P046 | 44 | MSM | L2b | 1 | 3 | 3 | 3 | 2 | 2 | 19 |
|  | P052 | 44 | MSM | L2b | 1 | 3 | 3 | 3 | 2 | 2 | 19 |
|  | P056 | 44 | MSM | L2b | 1 | 3 | 3 | 3 | 2 | 2 | 19 |
|  | P062 | 44 | MSM | L2b | 1 | 3 | 3 | 3 | 2 | 2 | 19 |
|  | P067 | 44 | MSM | L2b | 1 | 3 | 3 | 3 | 2 | 2 | 19 |
|  | P068 | 44 | MSM | L2b | 1 | 3 | 3 | 3 | 2 | 2 | 19 |
|  | P076 | 44 | MSM | L2b | 1 | 3 | 3 | 3 | 2 | 2 | 19 |
|  | P078 | 44 | MSM | L2 | 1 | 3 | 3 | 3 | 2 | 2 | 19 |
|  | P083 | 44 | MSM | L2b | 1 | 3 | 3 | 3 | 2 | 2 | 19 |
|  | P089 | 44 | MSM | L2b | 1 | 3 | 3 | 3 | 2 | 2 | 19 |
|  | P091 | 44 | MSM | L2b | 1 | 3 | 3 | 3 | 2 | 2 | 19 |
| ^a^ Hetero, heterosexual; MSM , men who have sex with men | | | | | | | | | | | |
