# Supplementary material for: Does typing of Chlamydia trachomatis using housekeeping multilocus sequence typing reveal different sexual networks among heterosexuals and men who have sex with men?
Source: BMC Infect Dis. 2016 Apr 18;16:162. doi: 10.1186/s12879-016-1486-2 (PMC4836166; doi:10.1186/s12879-016-1486-2)
Supplement: Additional file 2: Table S2. — Hr-MLST-6 data of the 187 successfully typed samples. (DOCX 53 kb) [file 12879_2016_1486_MOESM2_ESM.docx]

**Supplementary Table 2. Hr-MLST-6 data of the 187 successfully typed samples.**

Coding is according to the hr-MLST-6 database (http:// mlstdb.bmc.uu.se/). The samples are sorted by cluster and sequence type.

| **Cluster name** | **ID** | **Sequence type** | **Sexgroup^a^** | ***ompA* genovar** | ***ompA*** | **CT046** | **CT058** | **CT144** | **CT172** | **CT682** |
| --- | --- | --- | --- | --- | --- | --- | --- | --- | --- | --- |
| Cluster I | P007 | 108c | MSM | J | 53 | 29 | 8 | 5 | 3 | 6 |
|  | P016 | 108c | MSM | J | 53 | 29 | 8 | 5 | 3 | 6 |
|  | P017 | 108c | MSM | J | 53 | 29 | 8 | 5 | 3 | 6 |
|  | P028 | 108c | MSM | J | 53 | 29 | 8 | 5 | 3 | 6 |
|  | P051 | 108c | MSM | J | 53 | 29 | 8 | 5 | 3 | 6 |
|  | P054 | 108c | MSM | J | 53 | 29 | 8 | 5 | 3 | 6 |
|  | P065 | 108c | MSM | J | 53 | 29 | 8 | 5 | 3 | 6 |
|  | P072 | 108c | MSM | J | 53 | 29 | 8 | 5 | 3 | 6 |
|  | P075 | 108c | MSM | J | 53 | 29 | 8 | 5 | 3 | 6 |
|  | P008 | 307 | MSM | J | 53 | 29 | 8 | 5 | 3 | 58 |
|  | P071 | 307 | MSM | J | 53 | 29 | 8 | 5 | 3 | 58 |
|  | P038 | 345 | MSM | J | 53 | 74 | 8 | 5 | 3 | 6 |
|  | P049 | 345 | MSM | J | 53 | 74 | 8 | 5 | 3 | 6 |
| Cluster II | P026 | 33 | MSM | G | 8 | 10 | 8 | 5 | 3 | 6 |
|  | P037 | 33 | MSM | G | 8 | 10 | 8 | 5 | 3 | 6 |
|  | P040 | 33 | MSM | G | 8 | 10 | 8 | 5 | 3 | 6 |
|  | P055 | 33 | MSM | G | 8 | 10 | 8 | 5 | 3 | 6 |
|  | P059 | 33 | MSM | G | 8 | 10 | 8 | 5 | 3 | 6 |
|  | P074 | 33 | MSM | G | 8 | 10 | 8 | 5 | 3 | 6 |
|  | P006 | 52 | MSM | G | 8 | 20 | 8 | 5 | 3 | 6 |
|  | P014 | 52 | MSM | G | 8 | 20 | 8 | 5 | 3 | 6 |
|  | P020 | 52 | MSM | G | 8 | 20 | 8 | 5 | 3 | 6 |
|  | P023 | 52 | MSM | G | 8 | 20 | 8 | 5 | 3 | 6 |
|  | P025 | 52 | MSM | G | 8 | 20 | 8 | 5 | 3 | 6 |
|  | P030 | 52 | MSM | G | 8 | 20 | 8 | 5 | 3 | 6 |
|  | P031 | 52 | MSM | G | 8 | 20 | 8 | 5 | 3 | 6 |
|  | P033 | 52 | MSM | G | 8 | 20 | 8 | 5 | 3 | 6 |
|  | P048 | 52 | MSM | G | 8 | 20 | 8 | 5 | 3 | 6 |
|  | P061 | 52 | MSM | G | 8 | 20 | 8 | 5 | 3 | 6 |
|  | P066 | 52 | MSM | G | 8 | 20 | 8 | 5 | 3 | 6 |
|  | P069 | 52 | MSM | G | 8 | 20 | 8 | 5 | 3 | 6 |
|  | P070 | 52 | MSM | G | 8 | 20 | 8 | 5 | 3 | 6 |
|  | P080 | 52 | MSM | G | 8 | 20 | 8 | 5 | 3 | 6 |
|  | P082 | 52 | MSM | G | 8 | 20 | 8 | 5 | 3 | 6 |
|  | P086 | 52 | MSM | G | 8 | 20 | 8 | 5 | 3 | 6 |
|  | P087 | 52 | MSM | G | 8 | 20 | 8 | 5 | 3 | 6 |
|  | P050 | 301 | MSM | G | 8 | 20 | 8 | 5 | 1 | 6 |
|  | P009 | 303 | MSM | G | 8 | 20 | 8 | 5 | 3 | 34 |
|  | P024 | 304 | MSM | G | 8 | 20 | 8 | 5 | 4 | 6 |
|  | P085 | 353 | MSM | G | 8 | 81 | 8 | 5 | 3 | 6 |
| Cluster III | P106 | 100b | Hetero | I | 36 | 10 | 5 | 12 | 7 | 18 |
|  | P119 | 100b | Hetero | I | 36 | 10 | 5 | 12 | 7 | 18 |
|  | P165 | 100b | Hetero | I | 36 | 10 | 5 | 12 | 7 | 18 |
|  | P139 | 100c | Hetero | I | 37 | 10 | 5 | 12 | 7 | 18 |
|  | P096 | 101 | Hetero | I | 36 | 38 | 5 | 12 | 7 | 18 |
|  | P142 | 135a | Hetero | J | 20 | 10 | 5 | 12 | 4 | 18 |
|  | P123 | 135b | Hetero | I | 36 | 10 | 5 | 12 | 4 | 18 |
|  | P181 | 135b | Hetero | I | 36 | 10 | 5 | 12 | 4 | 18 |
|  | P131 | 272 | Hetero | I | 36 | 10 | 5 | 12 | 24 | 18 |
|  | P099 | 517 | Hetero | I | 36 | 38 | 5 | 12 | 3 | 18 |
|  | P116 | 517 | Hetero | I | 36 | 38 | 5 | 12 | 3 | 18 |
| Cluster IV | P057 | 12d | MSM | F | 24 | 5 | 19 | 7 | 1 | 4 |
|  | P098 | 12d | Hetero | F | 24 | 5 | 19 | 7 | 1 | 4 |
|  | P103 | 12d | Hetero | F | 24 | 5 | 19 | 7 | 1 | 4 |
|  | P108 | 12d | Hetero | F | 24 | 5 | 19 | 7 | 1 | 4 |
|  | P120 | 12d | Hetero | F | 24 | 5 | 19 | 7 | 1 | 4 |
|  | P124 | 12d | Hetero | F | 24 | 5 | 19 | 7 | 1 | 4 |
|  | P132 | 12d | Hetero | F | 24 | 5 | 19 | 7 | 1 | 4 |
|  | P135 | 12d | Hetero | F | 24 | 5 | 19 | 7 | 1 | 4 |
|  | P138 | 12d | Hetero | F | 24 | 5 | 19 | 7 | 1 | 4 |
|  | P151 | 12d | Hetero | F | 24 | 5 | 19 | 7 | 1 | 4 |
|  | P156 | 12d | Hetero | F | 24 | 5 | 19 | 7 | 1 | 4 |
|  | P171 | 12d | Hetero | F | 24 | 5 | 19 | 7 | 1 | 4 |
|  | P185 | 12d | Hetero | F | 24 | 5 | 19 | 7 | 1 | 4 |
|  | P160 | 13b | Hetero | F | 24 | 5 | 19 | 15 | 1 | 4 |
|  | P041 | 90 | MSM | F | 24 | 5 | 19 | 1 | 1 | 4 |
|  | P097 | 90 | Hetero | F | 24 | 5 | 19 | 1 | 1 | 4 |
|  | P035 | 91a | MSM | F | 24 | 5 | 19 | 5 | 2 | 4 |
|  | P169 | 110 | Hetero | F | 24 | 1 | 2 | 7 | 2 | 4 |
|  | P032 | 148a | MSM | F | 24 | 5 | 19 | 7 | 2 | 4 |
|  | P175 | 148a | Hetero | F | 24 | 5 | 19 | 7 | 2 | 4 |
|  | P154 | 240 | Hetero | F | 24 | 5 | 19 | 12 | 1 | 4 |
|  | P100 | 450 | Hetero | F | 24 | 1 | 19 | 7 | 2 | 4 |
|  | P172 | 466 | Hetero | F | 24 | 5 | 19 | 7 | 21 | 4 |
| Cluster V | P001 | 109 | MSM | D | 1 | 5 | 20 | 5 | 2 | 34 |
|  | P002 | 109 | MSM | D | 1 | 5 | 20 | 5 | 2 | 34 |
|  | P003 | 109 | MSM | D | 1 | 5 | 20 | 5 | 2 | 34 |
|  | P010 | 109 | MSM | D | 1 | 5 | 20 | 5 | 2 | 34 |
|  | P012 | 109 | MSM | D | 1 | 5 | 20 | 5 | 2 | 34 |
|  | P021 | 109 | MSM | D | 1 | 5 | 20 | 5 | 2 | 34 |
|  | P029 | 109 | MSM | D | 1 | 5 | 20 | 5 | 2 | 34 |
|  | P034 | 109 | MSM | D | 1 | 5 | 20 | 5 | 2 | 34 |
|  | P039 | 109 | MSM | D | 1 | 5 | 20 | 5 | 2 | 34 |
|  | P044 | 109 | MSM | D | 1 | 5 | 20 | 5 | 2 | 34 |
|  | P047 | 109 | MSM | D | 1 | 5 | 20 | 5 | 2 | 34 |
|  | P053 | 109 | MSM | D | 1 | 5 | 20 | 5 | 2 | 34 |
|  | P063 | 109 | MSM | D | 1 | 5 | 20 | 5 | 2 | 34 |
|  | P064 | 109 | MSM | D | 1 | 5 | 20 | 5 | 2 | 34 |
|  | P073 | 109 | MSM | D | 1 | 5 | 20 | 5 | 2 | 34 |
|  | P077 | 109 | MSM | D | 1 | 5 | 20 | 5 | 2 | 34 |
|  | P084 | 109 | MSM | D | 1 | 5 | 20 | 5 | 2 | 34 |
|  | P090 | 109 | MSM | D | 1 | 5 | 20 | 5 | 2 | 34 |
|  | P036 | 194 | MSM | D | 1 | 5 | 6 | 5 | 2 | 34 |
|  | P058 | 194 | MSM | D | 1 | 5 | 6 | 5 | 2 | 34 |
|  | P088 | 194 | MSM | D | 1 | 5 | 6 | 5 | 2 | 34 |
|  | P060 | 318 | MSM | D | 1 | 45 | 20 | 5 | 2 | 34 |
|  | P022 | 324 | MSM | D | 1 | 55 | 20 | 5 | 2 | 34 |
| Cluster VI | P112 | 16 | Hetero | E | 6 | 7 | 19 | 14 | 2 | 1 |
|  | P045 | 56a | MSM | E | 6 | 1 | 19 | 7 | 2 | 1 |
|  | P081 | 56a | MSM | E | 6 | 1 | 19 | 7 | 2 | 1 |
|  | P092 | 56a | Hetero | E | 6 | 1 | 19 | 7 | 2 | 1 |
|  | P093 | 56a | Hetero | E | 6 | 1 | 19 | 7 | 2 | 1 |
|  | P095 | 56a | Hetero | E | 6 | 1 | 19 | 7 | 2 | 1 |
|  | P118 | 56a | Hetero | E | 6 | 1 | 19 | 7 | 2 | 1 |
|  | P126 | 56a | Hetero | E | 6 | 1 | 19 | 7 | 2 | 1 |
|  | P133 | 56a | Hetero | E | 6 | 1 | 19 | 7 | 2 | 1 |
|  | P155 | 56a | Hetero | E | 6 | 1 | 19 | 7 | 2 | 1 |
|  | P170 | 59 | Hetero | E | 6 | 7 | 19 | 7 | 2 | 1 |
|  | P104 | 153 | Hetero | E | 6 | 35 | 19 | 7 | 2 | 1 |
|  | P125 | 153 | Hetero | E | 6 | 35 | 19 | 7 | 2 | 1 |
|  | P127 | 171 | Hetero | E | 6 | 49 | 19 | 7 | 2 | 1 |
|  | P166 | 171 | Hetero | E | 6 | 49 | 19 | 7 | 2 | 1 |
|  | P111 | 453 | Hetero | E | 6 | 1 | 19 | 7 | 21 | 1 |
|  | P121 | 498 | Hetero | E | 6 | 24 | 19 | 14 | 2 | 1 |
|  | P157 | 504 | Hetero | E | 6 | 47 | 19 | 7 | 2 | 1 |
|  | P167 | 513 | Hetero | E | 6 | 71 | 19 | 7 | 2 | 1 |
|  | P186 | 513 | Hetero | E | 6 | 71 | 19 | 7 | 2 | 1 |
| Cluster VII | P102 | 3 | Hetero | E | 6 | 1 | 2 | 6 | 2 | 2 |
|  | P128 | 3 | Hetero | E | 6 | 1 | 2 | 6 | 2 | 2 |
|  | P130 | 3 | Hetero | E | 6 | 1 | 2 | 6 | 2 | 2 |
|  | P136 | 3 | Hetero | E | 6 | 1 | 2 | 6 | 2 | 2 |
|  | P141 | 3 | Hetero | E | 6 | 1 | 2 | 6 | 2 | 2 |
|  | P158 | 3 | Hetero | E | 6 | 1 | 2 | 6 | 2 | 2 |
|  | P159 | 3 | Hetero | E | 6 | 1 | 2 | 6 | 2 | 2 |
|  | P164 | 3 | Hetero | E | 6 | 1 | 2 | 6 | 2 | 2 |
|  | P174 | 3 | Hetero | E | 6 | 1 | 2 | 6 | 2 | 2 |
|  | P180 | 3 | Hetero | E | 6 | 1 | 2 | 6 | 2 | 2 |
|  | P152 | 172 | Hetero | E | 6 | 1 | 2 | 7 | 2 | 2 |
|  | P168 | 305 | Hetero | E | 6 | 25 | 2 | 6 | 2 | 2 |
|  | P161 | 448 | Hetero | E | 6 | 1 | 2 | 11 | 2 | 2 |
|  | P177 | 509 | Hetero | E | 6 | 71 | 2 | 1 | 2 | 2 |
|  | P173 | 510 | Hetero | E | 6 | 71 | 2 | 6 | 2 | 2 |
| Cluster VIII | P078 | 58a | MSM | L2 | 22 | 27 | 13 | 17 | 13 | 28 |
|  | P011 | 58b | MSM | L2b | 28 | 27 | 13 | 17 | 13 | 28 |
|  | P015 | 58b | MSM | L2b | 28 | 27 | 13 | 17 | 13 | 28 |
|  | P018 | 58b | MSM | L2b | 28 | 27 | 13 | 17 | 13 | 28 |
|  | P019 | 58b | MSM | L2b | 28 | 27 | 13 | 17 | 13 | 28 |
|  | P027 | 58b | MSM | L2b | 28 | 27 | 13 | 17 | 13 | 28 |
|  | P042 | 58b | MSM | L2b | 28 | 27 | 13 | 17 | 13 | 28 |
|  | P046 | 58b | MSM | L2b | 28 | 27 | 13 | 17 | 13 | 28 |
|  | P052 | 58b | MSM | L2b | 28 | 27 | 13 | 17 | 13 | 28 |
|  | P056 | 58b | MSM | L2b | 28 | 27 | 13 | 17 | 13 | 28 |
|  | P062 | 58b | MSM | L2b | 28 | 27 | 13 | 17 | 13 | 28 |
|  | P067 | 58b | MSM | L2b | 28 | 27 | 13 | 17 | 13 | 28 |
|  | P068 | 58b | MSM | L2b | 28 | 27 | 13 | 17 | 13 | 28 |
|  | P076 | 58b | MSM | L2b | 28 | 27 | 13 | 17 | 13 | 28 |
|  | P083 | 58b | MSM | L2b | 28 | 27 | 13 | 17 | 13 | 28 |
|  | P089 | 58b | MSM | L2b | 28 | 27 | 13 | 17 | 13 | 28 |
|  | P091 | 58b | MSM | L2b | 28 | 27 | 13 | 17 | 13 | 28 |
|  | P013 | 143 | MSM | L2b | 28 | 44 | 13 | 17 | 13 | 28 |
| Residual group | P004 | 11 | MSM | D | 1 | 5 | 19 | 7 | 2 | 10 |
|  | P005 | 11 | MSM | D | 1 | 5 | 19 | 7 | 2 | 10 |
|  | P043 | 11 | MSM | D | 1 | 5 | 19 | 7 | 2 | 10 |
|  | P115 | 20a | Hetero | D | 2 | 10 | 4 | 1 | 4 | 17 |
|  | P137 | 27 | Hetero | G | 9 | 10 | 6 | 10 | 1 | 6 |
|  | P079 | 30 | MSM | K | 12 | 10 | 7 | 1 | 3 | 8 |
|  | P094 | 32 | Hetero | K | 12 | 10 | 7 | 1 | 4 | 8 |
|  | P149 | 35 | Hetero | D | 2 | 10 | 8 | 1 | 4 | 17 |
|  | P150 | 35 | Hetero | D | 2 | 10 | 8 | 1 | 4 | 17 |
|  | P176 | 35 | Hetero | D | 2 | 10 | 8 | 1 | 4 | 17 |
|  | P122 | 69 | Hetero | E | 6 | 5 | 19 | 6 | 2 | 2 |
|  | P113 | 74 | Hetero | B | 30 | 8 | 8 | 1 | 7 | 18 |
|  | P182 | 77b | Hetero | D | 31 | 5 | 19 | 7 | 2 | 37 |
|  | P183 | 97b | Hetero | H | 35 | 12 | 5 | 11 | 9 | 8 |
|  | P184 | 137 | Hetero | G | 8 | 10 | 8 | 22 | 4 | 6 |
|  | P147 | 165 | Hetero | H | 35 | 12 | 5 | 1 | 9 | 8 |
|  | P187 | 205b | Hetero | J | 20 | 10 | 6 | 22 | 4 | 8 |
|  | P144 | 220a | Hetero | K | 12 | 10 | 4 | 1 | 3 | 8 |
|  | P140 | 232 | Hetero | J | 20 | 5 | 19 | 1 | 4 | 18 |
|  | P143 | 232 | Hetero | J | 20 | 5 | 19 | 1 | 4 | 18 |
|  | P114 | 265 | Hetero | G | 10 | 10 | 4 | 1 | 3 | 7 |
|  | P179 | 270 | Hetero | G | 8 | 10 | 5 | 12 | 3 | 8 |
|  | P178 | 281a | Hetero | J | 20 | 10 | 8 | 1 | 4 | 18 |
|  | P153 | 281b | Hetero | J | 63 | 10 | 8 | 1 | 4 | 18 |
|  | P146 | 288 | Hetero | G | 8 | 10 | 8 | 22 | 7 | 57 |
|  | P148 | 338 | Hetero | E | 6 | 67 | 2 | 7 | 2 | 60 |
|  | P145 | 341 | Hetero | E | 6 | 70 | 57 | 7 | 2 | 1 |
|  | P107 | 395 | Hetero | D | 2 | 83 | 4 | 1 | 3 | 17 |
|  | P163 | 435 | Hetero | G | 9 | 8 | 6 | 10 | 1 | 6 |
|  | P162 | 449 | Hetero | E | 6 | 1 | 19 | 1 | 1 | 1 |
|  | P109 | 459 | Hetero | E | 6 | 5 | 19 | 6 | 21 | 2 |
|  | P110 | 462 | Hetero | E | 6 | 5 | 19 | 7 | 2 | 56 |
|  | P134 | 465 | Hetero | E | 6 | 5 | 19 | 7 | 14 | 2 |
|  | P101 | 482 | Hetero | G | 9 | 10 | 6 | 10 | 1 | 8 |
|  | P129 | 484 | Hetero | I | 37 | 10 | 7 | 1 | 3 | 5 |
|  | P117 | 502 | Hetero | E | 6 | 45 | 19 | 6 | 1 | 2 |
|  | P105 | 516 | Hetero | E | 6 | 5 | 19 | 5 | 2 | 2 |
| ^a^ Hetero, heterosexual; MSM , men who have sex with men | | | | | | | | | | |
